# Optimized maritime emergency resource allocation under dynamic demand

**DOI:** 10.1371/journal.pone.0189411

**Published:** 2017-12-14

**Authors:** Wenfen Zhang, Xinping Yan, Jiaqi Yang

**Affiliations:** 1 Intelligent Transportation System Research Center (ITSC), Wuhan University of Technology, Wuhan, China; 2 National Engineering Research Center for Water Transport Safety(WTSC),MoST Wuhan, China; 3 School of Transportation, Wuhan University of Technology, Wuhan, China; Beihang University, CHINA

## Abstract

Emergency resource is important for people evacuation and property rescue when accident occurs. The relief efforts could be promoted by a reasonable emergency resource allocation schedule in advance. As the marine environment is complicated and changeful, the place, type, severity of maritime accident is uncertain and stochastic, bringing about dynamic demand of emergency resource. Considering dynamic demand, how to make a reasonable emergency resource allocation schedule is challenging. The key problem is to determine the optimal stock of emergency resource for supplier centers to improve relief efforts. This paper studies the dynamic demand, and which is defined as a set. Then a maritime emergency resource allocation model with uncertain data is presented. Afterwards, a robust approach is developed and used to make sure that the resource allocation schedule performs well with dynamic demand. Finally, a case study shows that the proposed methodology is feasible in maritime emergency resource allocation. The findings could help emergency manager to schedule the emergency resource allocation more flexibly in terms of dynamic demand.

## Introduction

Maritime safety has attracted an increasing attention in recent decades. Maritime emergencies occur frequently, causing great damage to environment and society. For example, the accident that Oriental Star wrecked on June 1st, 2015, has raised the worldwide shock. Maritime emergency logistics management has emerged as a globally concerned theme as maritime accidents ubiquitously occur around the world. In practice, the difficulty of relief-demand management is rooted in the uncertainties[1]. Emergency resource demand, unlike business logistic of commodity demand, features uncertainty, which is dynamic demand. As the marine environment is complicated and changefully, the place, type, severity of maritime accident is uncertain and stochastic, bringing about dynamic demand of emergency resource. Considering dynamic demand, how to make a reasonable emergency resource allocation schedule is challenging.

Due to the sudden and high variation in the occurrence of emergency, the demand of emergency resource is known as a random variable [2]. At present, the maritime safety management relies on the historical data[3]. Time-varying relief demand is assumed highly correlated with the number of survivals, forecasting the time-vary relief demand associated with each affected area in a given time interval through updating the accumulated number of fatalities and relief demand approximation [1]. The expected demand and density function is identified by exponential smoothing method, TOPSIS and etc[2,4,5]. However, if the variation law of emergency demand is not obvious or the historical data about emergency demand is not available, the method of fuzzy logic and consistency-based linear programming could be used to integrate the associated influencing factors in the analysis of uncertainty caused by risk [6]. As is similar to dynamic demand, uncertainty is inherent to risk analysis. The qualitative framework and formalized risk assessment are popular in uncertainty maritime risk analysis [7, 8]. In such case, a set is introduced to describe the dynamic demand [9].

Disaster operations can be performed before or after disaster occurrence. The related research can be divided into pre-disaster operations (facility location-routing, stock) and post-disaster operations (relief distribution and transportation) [10]. Many researchers have studied on the logistic optimization and vehicle routing. Two-echelon logistics distribution model was established, a hybrid particle swarm optimization-genetic algorithm is proposed [11,12]. The relevant solution methods and objective functions of Multiple Depot Vehicle Routing Problem is summarized [13].

The pre-disaster operation is high related with response decisions. Therefore, preparedness and response planning are usually integrated [2, 5]. Several possible scenarios of these damages were drawn up and response actions for each of them were planned. The process of stocking warehouses consists of deciding the inventory level at every warehouse, while making sure the inventory level does not exceed the warehouse capacity [14]. The main influencing factor for optimizing resource allocation in post-disaster operation is time, an dynamic optimization model is introduced to find the best assignment/schedule of resources to areas. Multi-stage propositioning optimization model, real-time resource allocation model is developed, features several objectives: minimizing the fatalities, total cost, travel time, and maximizing the minimal satisfaction[15–17].The aforementioned dynamic demand is seem as relevant with time, the assumption is not appropriate in maritime emergency[18].

To deal with optimization problem with uncertain data, robust optimization is developed, which expresses the uncertain data in scenario or an uncertain set. Many robust approaches have been proposed in the field of emergency logistics.

There have been extensive related researches about the robust optimization method[19,20].The robust counterpart approach is applied to deal with uncertain convex program [21]. The dynamic demand can be expressed as an uncertain set. In terms of uncertain initial inventory, the robust optimization approach is applied in inventory control [9]. The efficiency of the method is demonstrated by simulation and practice [22, 23]. However, some dynamic demand may involve uncertain data. The corresponding robust optimization based on scenario focus on the variance and expected value of objective. The general framework for robustness is developed and the relative merits of robust optimization is discussed over sensitivity analysis and stochastic programming [24]. A multi-objective robust stochastic programming approach is developed for disaster relief logistics and multi-site production planning under uncertainty [25–27].

Despite the emergency logistic has increasingly drawn researcher’s attention, some research exhibits a number of drawbacks. In practice, the demand for maritime emergency is dynamic, with adjusting to frequent updated information, such as time, space, environment and etc. The dynamic demand analysis approach aforementioned is not appropriate in maritime emergency. In order to improve relief effort in maritime emergency, the emergency resource should be reserved in suppliers in a reasonable and effective way during the period of pre-disaster. It can be seen from the literature review that, the existing researches focused on the research during the period of post-disaster, with sufficient and systematic approach. Few papers study on the reasonable stock of emergency resource in advance. Moreover, the majority of model and approach ignore the uncertainty of demand.

Accordingly, this study focuses on the dynamic demand of maritime emergency resource. Taking the uncertain into consideration, a robust approach is introduced to determine the optimal stock of emergency resource.

The remainder of this paper is organized as follows. Firstly, the methodology of dynamic demand analysis is proposed, consists of three steps:1)dynamic demand forecasting,2)disaster area grouping,3)dynamic demand distribution. Then the robust optimized model is established for maritime emergency resource allocation during the preparedness phase, consists of three steps:1)problem definition,2)model establishing,3)robust model conversion. Finally, the dynamic demand analysis methodology and robust optimization model are applied in a case study and some interesting conclusions are drawn. The solution validation is demonstrated in S1 Appendix and the details of maritime accident in Shandong is depicted in S2 Appendix.

## Methodology

The methodology about maritime emergency allocation includes dynamic demand analysis and robust optimization. The dynamic demand of maritime emergency resource is analyzed in the first stage, to obtain the quantity and distribution of dynamic demand. Then, a robust optimization model is introduced to determine the optimal stock of emergency resource in the second stage.

### Dynamic demand analysis

Due to the uncertain maritime disasters, it is almost impossible to know the exact time and severity of an accident. It is very difficult to estimate the emergency resource in demand exactly in advance [28].However, combining the relief experience and forecasting approach, the fluctuation range of demand is available. In this study the dynamic demand can be expressed as a set $\left(d_{mi}^{0}-{\hat{d}}_{mi},d_{mi}^{0}+{\hat{d}}_{mi}\right)$. It’s worth noting that, the parameter $d_{mi}^{0}$ refers to the nominal demand of dynamic demand, namely the average demand. The parameter ${\hat{d}}_{mi}$ refers to the disturbance of dynamic demand, belongs to uncertain parameter.

The variation law of maritime emergency demand is not obvious, and the historical data of emergency demand is not available. Thus, it is not operational to estimate the dynamic demand accurately using historical data. We can analyze the influence factor of demand, and formalize a general methodology about dynamic demand. Yun-fei ai represents five criteria to identify the importance of water unit [5]. Zhang Di constructed a hierarchical structure for hazard identification model, taking into account both qualitative and quantitative criteria [29].

**Fig 1:**
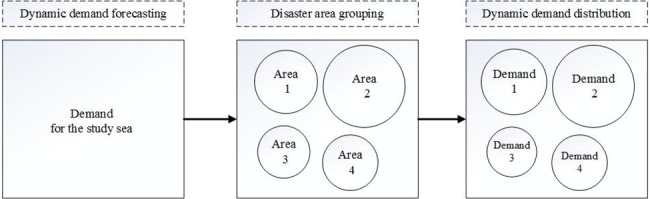
Scheme of dynamic demand analysis.

The distribution of maritime accidents appears to be dispersing. It’s infeasible to research the relief demand of each accident. Therefore, we could divide the study sea region into several disaster areas. Focusing on the disaster areas, the idea of "first whole then divide" is introduced to analysis the demand. Firstly, the total dynamic demand of the whole study area is forecasted, then group the disaster area. At last, the demand quantity of them is calculated. The procedure can be illustrated in Fig 1.

### Dynamic demand forecasting

In terms of the demand amount of maritime emergency resource, the transportation ministry of China has issued some documents about the general requirement for the sea area. According to the relevant regulations, the quantity and categories of emergency resource for a given sea area can be obtained by the Eq (1).

$$D_{m}=Q_{m}\times L_{t}\;m\in M$$ (1)

Where, *m* denotes the type of emergency resource; *D*_*m*_ denotes the dynamic demand quantity of the type k emergency resource for the study sea area; *Q*_*m*_ denotes the general requirement according to the relevant document; *L* denotes the comprehensive risk adjustment coefficient of the study sea area in certain time interval.

Taking the oil spill dispersant for example, the requirement for the terminal in sea area is shown in Table 1. The total requirement of oil spill dispersant for a given sea area can be calculated by summing up all the requirement of different grade terminal in the scope of the given sea area.

**Table 1:** The stock requirement of oil spill dispersant.

Terminal scale (million tons)	0.1~0.5	0.5~1	1~5	5 ~10	10 ~15	15 ~30	More than 30
Oil terminal oil spill dispersant (tons)	1	1.5	2	4	5.5	7.5	9
Other terminal oil spill dispersant (tons)	0.2	0.3	0.4	0.8	1.1	1.5	1.8

However, the requirement just refers to the general or basic demand. The comprehensive risk adjustment coefficient *L* represents the risk level of water unit, also indicates the demand extant of emergency resource, depending on the risk factors in the study area. As the time changes, the risk adjustment coefficient is dynamic. The corresponding relationship between *L* and *R* can be shown in Table 2.When the risk value *R* is between the grade limit as shown in Table 2, the adjustment coefficient *L* could be calculated by the linear interpolation method.

**Table 2:** The corresponding relationship between *L* and *R*.

Risk value of the sea area (*R*)	≤0.1	0.5	1	2	>3
Risk adjustment coefficient (*L*)	0.1	0.5	0.9	1.2	1.3

The water risk assessment method for China coastal area is proposed in the document, named Regulation on China maritime vessel allocation, issued in 2006.In this regulation, there are six criteria to determine the risk value of the coastal area as the table shown in Table 3. However, the threshold in Table 3 is only applicable in the China coastal area. In differ ent country, the threshold is different. The risk value of sea area is obtained by the Eq (2).

$$R=\frac{1}{6}\overset{6}{\underset{\theta =1}{\sum }}(R_{\theta }/K_{\theta })$$ (2)

Where,*θ* is the index of risk indicator, *R*_*θ*_ is the *θ*th risk indicator, *K*_*θ*_ is the threshold of the *θ*th risk indicator, which refers to the criterion risk level with the sea area is 50000m^2^.

**Table 3:** The criteria of risk value assessment.

Number of indicator	Indicator of risk assessment(R_θ_)	Threshold of indicator (K_θ_)
1	Traffic flow per year	280000
2	The number of tremendous and serious accident per year	16
3	The number of casualties per year	600
4	Throughput of cargo (thousand tons per year)	80000
5	Throughput of passenger (thousand person per year)	10000
6	Throughput of dangerous cargo (thousand tons per year)	12000

To eliminate the impact of occasional factors, it’s a good way to take the average value as the risk indicator from statistics during the recent years.

### Disaster area grouping

According to the spatial distribution of emergencies at sea, we aim to divide the study region into several disaster areas, namely disaster area grouping. The disaster area is represented by its center and scope. In this section, the center and the scope of each disaster area should be determined.

K-means clustering is a typical spatial clustering method. Because of its simplicity and good complexity. K-means clustering method is widely used in clustering analysis of large amounts of data. However, K-means clustering method is sensitive with the initial clustering center and number.The unreasonable selection of the clustering center and number will result in local optimum of the clustering scheme. Thus, the clustering result is unscientific, as well as the noise data will also affect the clustering result.

The minimum circle coverage is the drawing theory used to deal with the coverage of the column points in the plane geometry. The basic idea is to cover the set of points with the smallest circle and filter out the noise data to ensure that the largest blank area between the smallest circles. The minimum circle coverage has a good application prospect in information filtering, base location, algorithm design and so on. By maximizing the negative judgment area, the performance of filtering query is improved. Minimizing the coverage radius could help achieve the total distance shortest, between cluster center with all the data point. Searching for maritime disaster area is also maximizing the safety waters. So the minimum circular coverage method can be used in the initial division of the maritime disaster areas. The circle represents the maritime emergency gathering area. The center of the circle is the cluster center. The number of circles represents the number of clusters.

The K-means clustering method based on the minimum circle coverage could select the clustering number and the initial clustering center scientifically and intuitively, overcoming the blindness of the traditional K-means clustering method and the initial clustering center randomly selected method. The method is simple and feasible, which significantly reduces the number of iterations, and has strong adaptability to the irregularity of the study area boundary. Moreover, it can combine the actual distribution of the column points, remove the isolated point data. The clustering effect is obviously improved.

### Dynamic demand distribution

After disaster area grouping, we should determine the demand quantity of these disaster areas. This section develops the demand distribution method of emergency resource. The total demand of the whole study region is given. Therefore, the key problem is to identify the demand proportion of disaster area occupied in the whole study region, named demand weight. Entropy theory is an objective method of weight evaluation, to avoid human interference factors [1]. Based on the approximate criteria weights using the entropy theory, the demand of emergency resource for each disaster area can be obtained.

The following summarizes the main steps of dynamic demand distribution.

Step 1: Evaluation indicators for demand distribution

Suppose there are k disaster areas in the study region. Considering the actual situation in maritime emergency logistics, three indicators are proposed to determine the demand distribution, the number of maritime accidents, the number of casualties, and the number of damaged ships. The demand assessment matrix A is formatted by:

$$A=\left[\begin{array}{ccc} r_{11} & r_{12} & r_{13}\\ r_{21} & r_{22} & r_{23}\\ \ldots & \ldots & \ldots \\ r_{k1} & r_{k2} & r_{k3} \end{array}\right]$$ (3)

Where, the element *r*_*ij*,_ in which *i* = 1.2.*k*,*j* = 1.2.3 is the original value of the jth evaluation indicator in disaster area *i*. Considering the different measurement scales associated with these criteria, the original value should be standardized by Eq (4).

$$p_{ij}=r_{ij}/\sum_{i=1}^{k}r_{ij}\;j=1,2,3$$ (4)

*p*_*ij*_ represents the standardized evaluation value, then the standardized matrix can be obtained by Eq (5).

$$B=\left[\begin{array}{ccc} p_{11} & p_{12} & p_{13}\\ p_{21} & p_{22} & p_{23}\\ \ldots & \ldots & \ldots \\ p_{k1} & p_{k2} & p_{k3} \end{array}\right]$$ (5)

Step 2: Calculating the entropy value of evaluation indicator:

The entropy value of the jth evaluation indicator in demand distribution is given as:

$$e_{j}=-\ln{\left(k\right)}^{-1}\sum_{i=1}^{k}p_{ij}\ln p_{ij}$$ (6)

Step 3: Calculating the entropy weight of evaluation indicator

The entropy weight of the *j*th evaluation indicator in demand distribution is following.

$$E_{j}=\frac{1-e_{j}}{\sum_{j=1}^{3}\left(1-e_{j}\right)}$$ (7)

Step 4: Demand proportion identification of the disaster area

Sum up all the evaluation indexes for one disaster area with weight sum method, the demand weight of emergency resource for disaster area *i* is obtained.

$$w_{i}=\sum_{j=1}^{3}E_{j}p_{ij}$$ (8)

The greater of the weight for emergency resource demand, the greater share of the emergency resource demand in the area.

Step 5: The nominal dynamic demand

The nominal demand of type *m* emergency resource in disaster area *i* is derived by the following equation.

$$d_{mi}^{0}=D_{m}w_{i}$$ (9)

### Robust optimization model

#### Problem definition

**Fig 2:**
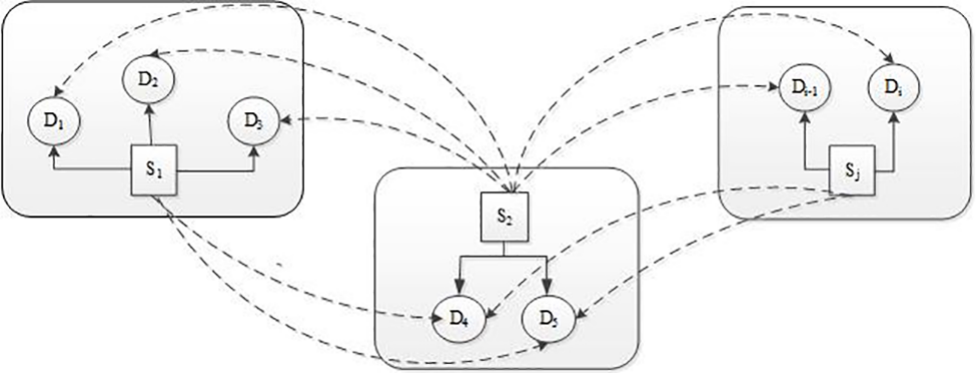
Maritime emergency resource allocation.

For the maritime emergency resource allocation under dynamic demand, the emergency resource demand of each disaster area is uncertain, varying in a set. This paper aims to determine the optimal quantity of emergency resource in each supplier, achieving the optimal of economic and effectiveness. A general overview of maritime emergency resource allocation can be described in Fig 2.

As is depicted in Fig 2,*S* is the emergency resource supplier center, *D* is the disaster area, The *i*th disaster area can be denoted by *D*_*i*_,the *j*th supplier can be denoted by *S*_*j*_. Once the maritime accident occurs, the emergency resource should be transit from the supplier center as near as possible. When the resource in the near supplier center is insufficient for the disaster area, the resource will transport from the distant supplier center. The priority of the supplier center is determined by the distance from the disaster area. For example, if the demand in disaster area *D*_*1*_ generates, the emergency resource should be transit from the supplier center *S*_*1*_, if the emergency resource is insufficient, the supplier center *S*_*2*_ will be the candidate. When all the supplier centers cannot meet the requirements, the unfulfilled demand resource will be purchased temporarily. The quantity of resource purchased is consider as the compensation demand.

Supposing there are altogether *I* disaster areas, *J* emergency resource supplier centers and M (*m* ∈ *M*) types emergency resource. The allocation model proposed aims to improve relief efficiency and economy, minimizing the sum of the maximum shortages in the affected areas.

To simplify the model, main assumptions of the proposed model are as follows:

1. Any supplier center can provide emergency resources to any disaster area. The maritime rescue is relatively difficult and time consuming. So, in practice, the transportation time is unlimited. In terms of major accidents rescue, emergency forces from the different regions should be joined together. So, the rescue regions are often not limit to the scope of the supplier center covered.
2. The emergency resources in one supplier center cannot be transferred to another. Usually, the resource supplier center is far away from another. When the resource in the supplier center is insufficient, the unfulfilled resource is purchased from the enterprise nearby or the agreement manufacturers, not from the other supplier center.

According to the experience of maritime emergency rescue, the time from the supplier center to the disaster, and the supplier centers capacity are known.

Some of the parameters have been mentioned above, in this section we only introduce the parameters used in the emergency resource allocation model. The following variables are defined for the model:

*V*_*j*_:the maximum storage capacity of supplier *S*_*j*_.

*t*_*mij*_:the transportation time for the type *m* emergency resource from supplier *S*_*j*_ to demand point *D*_*i*_.

*α*:the penalty coefficient of demand, it is noteworthy that the range for it is more than 1.

*d*_*mi*_:the dynamic demand for the type m emergency resource in disaster area *D*_*i*_. It takes value in the interval $\left(d_{mi}^{0}-{\hat{d}}_{mi},d_{mi}^{0}+{\hat{d}}_{mi}\right)$, with mean equals to the nominal value $d_{mi}^{0}$.

*x*_*mj*_:the quantity of type *m* emergency resource reserved in supplier *S*_*j*_.

*y*_*mij*_:the quantity of type *m* emergency resource assigned from supplier *S*_*j*_ to demand point *D*_*i*_.

*z*_*mj*_:the compensation quantity of type *m* emergency resource for supplier *S*_*j*_ When the emergency resource is insufficient.

#### Model

The optimal quantity of emergency resource will be determined by the model, aims to achieve the optimal of total time and loss.

The total time of emergency resource allocation refers to the total time of all emergency resource transportation from the supplier center to the disaster area. It can be obtained through the transportation volume multiplied by the transportation time.

The total loss of emergency resource allocation refers to the loss caused by emergency resources idle or shortage. As the loss of emergency resources shortage is more serious than idle, the demand compensation penalty coefficient is introduced to enlarge the shortage of emergency resources. The loss caused by outage could be measured by the compensation penalty coefficient multiplied by outage quantity.

Given the above assumptions and notations, the maritime emergency resource allocation model can be stated as follows:

$$\begin{array}{l} f_{1}=\min\sum_{m\in M}\sum_{i\in I}\sum_{j\in J}t_{mij}y_{mij}\\ f_{2}=\min\sum_{m\in M}\left\{\sum_{i\in I}\sum_{j\in J}\left(x_{mj}-y_{mij}\right)+\alpha \sum_{i\in I}z_{mi}\right\}\\ s.t.\left\{ \begin{array}{l} \sum_{m\in M}x_{mj}\leq V_{j},\forall j\\ \sum_{i\in I}y_{mij}\leq x_{mj},\forall m,j\\ \sum_{j\in J}y_{mij}+z_{mi}\geq d_{mi},\forall m,i\\ x_{mj},y_{mij},z_{mi}\geq 0,\forall m,i,j \end{array}\right. \end{array}$$ (10)

It is obviously that, the maritime emergency resource allocation model is a multi-objective stochastic programming. The first objective function is to minimize the total transportation time for emergency resource allocation. The second objective function is to minimize the total loss for emergency resource allocation. The first part is the loss due to excess reserve, could be denoted by quantity of idle emergency resource, the second part is the loss due to shortage, and could be denoted by the quantity of compensation emergency resource and the penalty coefficient. The first constraint ensures that the quantity of emergency resource reserved in the supplier center does not exceed its capacity. The second constraint guarantee the quantity of emergency resource assigned from supplier *S*_*j*_ cannot exceed the quantity reserved in it. The third constraint state the demand of disaster area should be satisfied.The last constraint ensure the variables are significant.

The total time of emergency resource allocation is related to the traffic flow of emergency resources, as well as the geographical distribution of supplier center and disaster area. The total loss of emergency resource allocation is related to the quantity of compensation, transportation and allocation. By the influence of dynamic demand, they restrict each other [30]. The total time and total loss of emergency resource allocation cannot achieve the optimal, there is a relationship consider as constraint and competition between them. Due to the dimensions and significance of the two goals is different, it is not available that the traditional linear weighting sum method is applied to convert two goals into one. Therefore, this paper retains the two objectives, and adopts the multi-objective optimization method to deal with the model in parallel.

There are some methods widely used to deal with the optimization problem with uncertain data, such as stochastic optimization, fuzzy optimization and robust optimization[6]. For the method of stochastic optimization, the probability distribution of random variables should be known. The fuzzy optimization method introduces some subjective factors, making the constraints flexible. In terms of maritime emergency resource, the demand of each disaster area is uncertain, varying in a set, the distribution of demand cannot be obtained accurately. Moreover, the emergency resources related to life rescue, resource allocation model constraints are strict. Therefore, the robust optimization method is the most applicable in the study of emergency resource allocation under dynamic demand among those approaches. It is good at solving the optimization problem with some data expressed as a set. Furthermore, the optimal solution could satisfy all the constraints absolutely.

#### Model conversion

In the model (10), the dynamic demand parameter *d*_*mi*_ is uncertain. Here,the variables *y*_*mij*_ and *z*_*mj*_ is forced to be an affine function of the parameter *d*_*mi*_. The affine function is given by:

$$\left\{ \begin{array}{l} y_{mij}=\omega_{mij}d_{mi}\\ z_{mi}=\varpi_{mi}d_{mi} \end{array}\right.\;\forall m,i,j$$ (11)

The model can be reformulated as follows:

$$\begin{array}{l} f_{1}=\min\sum_{m\in M}\sum_{i\in I}d_{mi}\left(\sum_{j\in J}\omega_{mij}t_{mij}\right)\\ f_{2}=\min\sum_{m\in M}\left\{\sum_{j\in J}x_{mj}+\sum_{i\in I}d_{mi}(\alpha \varpi_{mi}-\sum_{j\in J}\omega_{mij})\right\}\\ s.t.\left\{ \begin{array}{l} \sum_{m\in M}x_{mj}\leq V_{j},\forall j\\ \sum_{i\in I}\omega_{mij}d_{mi}\leq x_{mj},\forall m,j\\ \sum_{j\in J}\omega_{mij}+\varpi_{mi}\geq 1,\forall m,i\\ x_{mj},\omega_{mij},\varpi_{mi}\geq 0,\forall m,i,j \end{array}\right. \end{array}$$ (12)

Note that both objectives and constructions of the model involve uncertain data *d*_*mi*_, we develop robust optimization approach for them.

In order to measure the robustness of the objectives, we introduce a parameter Γ_0_, including but not limited to integer, taking value in the interval[0,*J*_*0*_], *J*_0_ = |*M*||*I*|. Where, *M* denotes the number of emergency resource types, *I* denotes the number of disaster areas. The role of the parameter Γ_0_ is to adjust the robustness of the whole uncertain demand. Accordingly, for *mi* ∈ *J*_0_, the quantity of emergency resource uncertain demand is ⌊Γ_0_⌋, with a uncertain demand changed by $(\Gamma_{0}-\left\lfloor \Gamma_{0}\right\rfloor ){\hat{d}}_{mi}$.

Therefore, the objectives of the model (12) becomes:

$$\begin{array}{l} f_{1}=\min\left\{\sum_{i\in I}\sum_{m\in M}d_{m}^{0}{}_{i}\left(\sum_{j\in J}\omega_{mij}t_{mij}\right)+\underset{\left\{\begin{array}{l} U_{0}\cup r_{0}|U_{0}\subseteq J_{0},\\ \left|U_{0}\right|=\left|\Gamma_{0}\right|,\\ r_{0}\in J_{0}\backslash U_{0} \end{array}\right\}}{\max}\left[\sum_{mi\in U_{0}}{\hat{d}}_{mi}\left(\sum_{j\in J}\omega_{mij}t_{mij}\right)+(\Gamma_{0}-\left\lfloor \Gamma_{0}\right\rfloor ){\hat{d}}_{r_{0}}\left(\sum_{j\in J}t_{r_{0}j}\omega_{r_{0}j}\right)\right]\right\}\\ f_{2}=\min\{\sum_{j\in J}\sum_{m\in M}x_{mj}+\sum_{i\in I}\sum_{m\in M}d_{mi}^{0}\left(\alpha \varpi_{mi}-\sum_{j\in J}\omega_{mij}\right)+\\ \;\underset{\left\{\begin{array}{l} U_{0}\cup r_{0}|U_{0}\subseteq J_{0},\\ \left|U_{0}\right|=\left|\Gamma_{0}\right|,\\ r_{0}\in J_{0}\backslash U_{0} \end{array}\right\}}{\max}\left[\sum_{mi\in U_{0}}{\hat{d}}_{mi}\left(\alpha \varpi_{mi}-\sum_{j\in J}\omega_{mij}\right)+\left(\Gamma_{0}-\left\lfloor \Gamma_{0}\right\rfloor \right){\hat{d}}_{r_{0}}\left(\alpha \varpi_{r_{0}}-\sum_{j\in J}\omega_{r_{0}j}\right)\right]\} \end{array}$$ (13)

Likewise, In terms of the type *m* emergency resource, we introduce the parameter Γ_*m*_, include but not limited to integer, taking value in the interval[0,*J*_*m*_], *J*_*m*_ = |*I*|, Γ_0_ = Γ_1_ + Γ_2_ + … + Γ_*m*_. The parameter Γ_*m*_ is to adjust the robustness of every uncertain demand. Accordingly, for *mi* ∈ *J*_*m*_, the quantity of type *m* emergency resource uncertain demand is ⌊Γ_*m*_⌋, with a uncertain demand changed by $(\Gamma_{m}-\left\lfloor \Gamma_{m}\right\rfloor ){\hat{d}}_{mi}$.

The second constraint of the model (12) becomes:

$$\sum_{i\in I}\omega_{mij}d_{mi}^{0}+\underset{\left\{\begin{array}{l} U_{m}\cup r_{m}|U_{m}\subseteq J_{m},\\ \left|U_{m}\right|=\left|\Gamma_{m}\right|,\\ r_{m}\in J_{m}\backslash U_{m} \end{array}\right\}}{\max}\left\{\sum_{i\in U_{m}}{\hat{d}}_{mi}\omega_{mij}+(\Gamma_{m}-\left\lfloor \Gamma_{m}\right\rfloor ){\hat{d}}_{mr_{m}}\omega_{mr_{m}j}\right\}\leq x_{mj},\forall m,j$$ (14)

In order to reformulate the model as a linear optimization, We intend to implement the methodology of robust counterpart proposed in Ben-Tal [19]and Dimitris Bertsimas [20](2004).

Given the following linear optimization model:

$$\begin{array}{l} \varphi =\max\sum_{mi\in J_{0}}{\hat{d}}_{mi}\left(\sum_{j\in J}t_{mij}\omega_{mij}\right)\xi_{mi}\\ s.t.\;\sum_{mi\in J_{0}}\xi_{mi}\leq \Gamma_{0}\\ \;0\leq \xi_{mi}\leq 1 \end{array}$$ (15)

Equals the dual of problem (15):

$$\begin{array}{l} \;\min\;\Gamma_{0}\gamma +\sum_{mi\in J_{0}}\lambda_{mi}\\ s.t.\;\gamma +\lambda_{mi}\geq {\hat{d}}_{mi}\left(\sum_{j\in J}t_{mij}\omega_{mij}\right)\;\forall mi\in J_{0}\\ \;\lambda_{mi}\geq 0,\;\forall mi\in J_{0}\\ \;\gamma \geq 0. \end{array}$$ (16)

By strong duality, the linear optimization model (15) is equal to the objective function value of linear optimization model (16).Through transforming, the maritime emergency resource allocation model can be reformulated as below:

$$\begin{array}{l} f_{1}=\min\left\{\sum_{i\in I}\sum_{m\in M}d_{m}^{0}{}_{i}\left(\sum_{j\in J}t_{mij}\omega_{mij}\right)+\Gamma_{0}\gamma +\sum_{mi\in J_{0}}\lambda_{mi}\right\}\\ f_{2}=\min\{\sum_{j\in J}\sum_{m\in M}x_{mj}+\sum_{i\in I}\sum_{m\in M}d_{mi}^{0}\left(\alpha \varpi_{mi}-\sum_{j\in J}\omega_{mij}\right)+\Gamma_{0}\gamma^{'}+\sum_{mi\in J_{0}}\lambda_{mi}^{'}\}\\ s.t.\left\{ \begin{array}{l} \sum_{m\in M}x_{mj}\leq V_{j},\forall j\\ \sum_{i\in I}\omega_{mij}d_{mi}^{0}+\Gamma_{m}\varsigma_{mj}+\sum_{i\in J_{m}}\tau_{mij}\leq x_{mj},\forall m,j\\ \sum_{j\in J}\omega_{mij}+\varpi_{mi}\geq 1,\forall m,i\\ \varsigma_{mj}+\tau_{mij}\geq {\hat{d}}_{mi}\omega_{mij}\;\forall mi\in J_{m}\\ \gamma +\lambda_{mi}\geq {\hat{d}}_{mi}\left(\sum_{j\in J}t_{mij}\omega_{mij}\right)\;\forall mi\in J_{0}\\ \gamma^{'}+\lambda_{mi}^{'}\geq {\hat{d}}_{mi}\left(\alpha \varpi_{mi}-\sum_{j\in J}\omega_{mij}\right)\;\forall mi\in J_{0}\\ x_{mj},\omega_{mij},\varpi_{mi},\varsigma_{mj},\tau_{mij},\gamma ,\lambda_{mi},\gamma^{'},\lambda_{mi}^{'}\geq 0,\forall m,i,j \end{array}\right. \end{array}$$ (17)

Comparing the model after formulation with the original model, in order to solve the decision variables, many new parameters raise up. The parameters $\varsigma_{mj},\tau_{mij},\gamma ,\lambda_{mi},\gamma^{'},\lambda_{mi}^{'}$ are generated by dual transformation, belong to auxiliary variable, having no practical meaning.

## Case study

### Data acquisition

By 2016, there are 476 terminals in Shandong maritime area in China (simplified as Shandong), among these, 214 terminals are more than ten thousand tons.The terminals distribution of different grade is shown in Table 4.The data comes from China port statistical yearbook. For example, as the Table 4 shows, there are 63 oil terminals, the scale is between 1000 to 5000 tons.

**Table 4:** The terminal in Shandong.

Grade of terminal scale (ten thousand tons)	0.1~0.5	0.5~1	1~5	5 ~10	10 ~15	15 ~30	≥30
Oil terminal	63	10	16	16	6	2	2
Other terminal	81	87	94	43	45	6	5

The risk indicator statistics in Shandong can be shown during 2012–2014, As shown in Table 5. To eliminate the impact of occasional factors, it’s available to take the average value as the risk indicator from statistics during the past three years[31].The standard risk value in the table is the result after standard transformation of the risk factors. In the calculation of standard risk value, the area conversion coefficient should be introduced. As Shandong sea area is 159,500 square kilometers and the standard sea area is 50,000 square kilometers, the area conversion coefficient is 5 / 15.95 = 0.3135. Therefore, the standard risk factor is the product of the conversion coefficient of this area multiplied by the average value of the risk factors for the past three years.

**Table 5:** The risk indicator of Shandong.

Indicator of risk assessment(*R*_*θ*_)	2012	2013	2014	Average value	Standard value of indicator
Traffic flow per year	162119	162120	162000	162080	50812
The quantity of tremendous and serious accident per year	22	28	28	26	8.1
The quantity casualties per year	1260	1741	890	1297	406
Throughput of cargo(thousand tons per year)	100000	89000	88500	92500	28998
Throughput of passenger(thousand person per year)	2973	2581	2604	2719	853
Throughput of dangerous cargo(thousand tons per year)	17100	22466	23595	21052	6600

There are 87 maritime accidents in Shandong. Find out the location coordinates of the maritime accidents, depicted by Supporting Information *S1* Appendix, including the longitude and latitude data, and draw the maritime accidents points on the map by ArcGis software.

### Dynamic demand analysis

#### Dynamic demand forecasting of Shandong

According to Tables 1 and 4, the general requirement of oil spill dispersant in Shandong can be calculated as 421.8 tons.

Put the standard value of indicator as shown in Table 5 and the threshold of indicator as shown in Table 3 into the risk value calculation Eq (2),the risk value of Shandong is estimated:

$$R=\frac{1}{6}\overset{6}{\underset{i=1}{\sum }}(R_{i}/K_{i})=1.89$$ (18)

According to the corresponding relationship between *L* and *R* (Table 2),the risk adjustment coefficient can be calculated as L = 1.167.Thus, the oil spill dispersant demand of Shandong is D = 1.167×421.8 = 492.2 tons.

#### Disaster area grouping

Based on the minimum circular coverage and spatial clustering method, the disaster areas grouping steps are as follows:

Step 1:Plot the spatial distribution map of maritime accidents. Find out the location coordinates of the maritime accidents in the study area, including the longitude and latitude data, and draw the maritime accidents points on the map by ArcGis software. Then, the spatial distribution map of maritime accidents is formed.

Step 2: Draw circle to cover the accidents points. Based on the minimum circle coverage theory, draw several unequal size circles to cover the accidents points in the map, filtering event noise. The circles should be as small as possible, to achieve the largest of the blank area outside the circle and the highest of accident points concentration. The number of circle is the cluster number. The center of circle is consider as the initial clustering center.

Step 3:Identification of disaster area

①Calculate the Euclidean distance between each accident point *x* to the clustering center,and put the accident point into the group closest to it.
②Update the cluster center.
③Calculate the square error criterion function.
④Repeat step ①-③ until the criterion function get convergence.

In terms of maritime accidents distribution map in Shandong, 8 unequal size circles are drawn to cover the accidents points, filtering event noise. The circles should be as small as possible, to achieve the largest of the blank area outside the circle and the highest of accident points concentration. 8 circles are formed P_1_-P_8_. The center of circle P_1_-P_8_ is consider as the initial clustering center.

By the method of K-means, the group of accident points is adjusted, as well as the cluster center. The final scope of disaster areas can be determined.The location coordinates of 8 cluster center is shown in Table 6, as well as the number of casualties and damaged ships.

**Table 6:** The disaster areas in Shandong.

Disaster area	Coordinates of disaster area center(E,N)	The number of accidents	The number of casualties	The number of damaged Ships
P_1_	(119.57, 38.16)	10	49	7
P_2_	(121.17, 38.12)	11	91	12
P_3_	(122.41, 37.94)	13	65	9
P_4_	(122.98, 37.18)	8	82	9
P_5_	(122.46, 36.61)	12	92	12
P_6_	(120.53,35.85)	11	96	11
P_7_	(122.88, 35.71)	8	54	4
P_8_	(123.99, 35.28)	7	28	1

#### Dynamic demand distribution

After disaster area grouping, apply the Entropy theory method, the dynamic demand distribution of Shandong maritime area can be determined. The standard demand assessment matrix A of 8 disaster areas is formatted as Eq (19).

$$P={\left(p_{ij}\right)}_{m\times k}=\left[\begin{array}{ccc} 0.125 & 0.088 & 0.108\\ 0.138 & 0.163 & 0.185\\ 0.163 & 0.117 & 0.138\\ 0.1 & 0.147 & 0.138\\ 0.15 & 0.165 & 0.185\\ 0.138 & 0.172 & 0.169\\ 0.1 & 0.097 & 0.062\\ 0.088 & 0.050 & 0.015 \end{array}\right]$$ (19)

By Eq (6),the entropy value of evaluation indicator can be calculated, the entropy value for the number of maritime accidents, the number of casualties, the number of damaged ships are *e*_*1*_ = 0.988, *e*_*2*_ = 0.969, *e*_*3*_ = 0.935 respectively.

By Eq (7),the entropy weight of evaluation indicator for the number of maritime accidents, the number of casualties, the number of damaged ships are *E*_*1*_ = 0.111, *E*_*2*_ = 0.287, *E*_*3*_ = 0.602 respectively.

Sum up all the evaluation indexes for one disaster area with weight sum method, the demand weight ***w***_***i***_ for the 8 disaster areas are obtained by the Eq (8),as is shown in Table 7.

**Table 7:** Demand weight for the 8 disaster areas.

Disaster area	P_1_	P_2_	P_3_	P_4_	P_5_	P_6_	P_7_	P_8_
Demand weight *w*_*i*_	0.104	0.173	0.135	0.137	0.175	0.167	0.076	0.033

The nominal demand *d*_*i*_ of oil dispersant for 8 disaster area is derived by *d*_*mi*_ = *D*_*m*_*w*_*i*_, as shown in Table 8. when the demand disturb level is 2%, 5%, 10%, the dynamic demand for the 8 disaster area is shown in Table 9.

**Table 8:** Nominal demand for disaster areas.

Disaster area	P_1_	P_2_	P_3_	P_4_	P_5_	P_6_	P_7_	P_8_
Oil dispersant demand	50.7	84.4	65.8	66.8	85.4	81.4	37.1	16.1

**Table 9:** Dynamic demand for disaster areas.

Disaster area		P_1_	P_2_	P_3_	P_4_	P_5_	P_6_	P_7_	P_8_
disturbance 5%	Upper limit	53.2	88.6	69.1	70.1	89.7	85.5	38.9	16.9
	Lower limit	48.2	80.2	62.5	63.5	81.1	77.3	35.2	15.3
disturbance 10%	Upper limit	55.8	92.8	72.4	73.5	93.9	89.5	40.8	17.7
	Lower limit	45.6	75.9	59.2	60.1	76.9	73.3	33.4	14.5
disturbance 20%	Upper limit	60.8	101.3	78.9	80.2	102.5	97.7	44.5	19.3
	Lower limit	40.6	67.5	52.6	53.4	68.3	65.1	29.7	12.9

From the process of dynamic demand forecast and spatial distribution of emergency resources in Shandong maritime area. It can be seen that the total quantity of emergency resources demand in sea area will change dynamically over time, as well as the scope and the weight of disaster areas maritime emergencies. Therefore, the dynamic emergency resources demand of disaster area is also constantly adjusted and updated.

**Fig 3:**
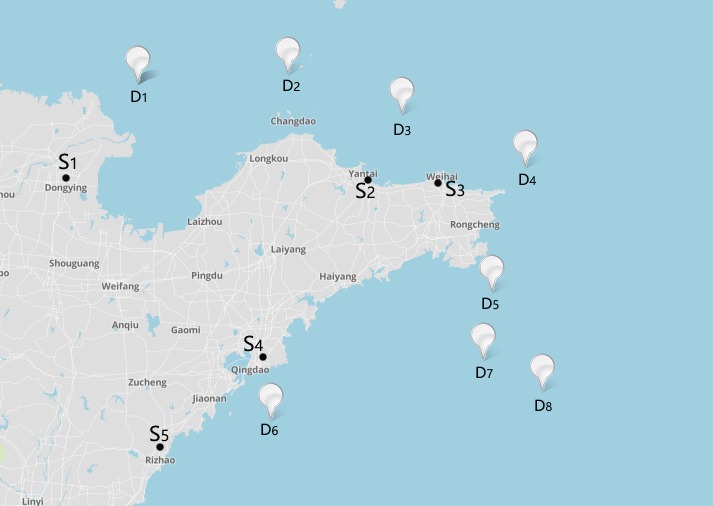
Distribution of suppliers and disaster areas.

According to the document named National water traffic safety supervision and rescue system layout planning, there are 5 maritime emergency resource supplier center located in Shandong coast, Jinan, Yantai, Weihai, Qingdao, and Rizhao, denoted by *S*_*1*_, *S*_*2*_, *S*_*3*_, *S*_*4*_, *S*_*5*_ in the map. In the last section, the disaster areas can be determined, denoted by *D*_*1*_, *D*_*2*_, *D*_*3*_, *D*_*4*_, *D*_*5*_, *D*_*6*_, *D*_*7*_, *D*_*8*_. Using the position data of disaster areas and supplier center, the distribution map of supplier centers and disaster areas could be drawn on the map from https://www.mapbox.com/. The maritime emergency supplier centers and disaster areas map of Shandong is shown in Fig 3.

The location data of supplier center and disaster area is put into the map software, by the use of distance measurement tool, the distance between emergency resource supplier center and the disaster area center is shown in Table 10.

**Table 10:** Distance between supplier center and the disaster area center (kilometers).

Supplier center Disaster area	*S*_*1*_	*S*_*2*_	*S*_*3*_	*S*_*4*_	*S*_*5*_
D_1_	105	215	353	630	745
D_2_	209	72	205	473	562
D_3_	322	97	103	386	500
D_4_	405	165	40	290	383
D_5_	508	255	72	210	295
D_6_	702	480	292	50	89
D_7_	575	355	177	228	305
D_8_	647	430	245	335	400

The maritime accidents statistics show that, most of the maritime emergencies occurs in the range of 10–30 nautical miles from the sea shore, and emergency resources supplier center is mainly built in the port terminal. Therefore, emergency resources from the supplier center to the accident area mainly by sea. The average speed of the ship is 35kn, so the average speed of emergency resources from the supplier center to the accident area is 64.82km /h. Table 10 provides the distance from the supplier center to the accident center, the transportation time is shown in Table 11.

**Table 11:** The transportation time (hour).

Supplier center Disaster area	*S*_*1*_	*S*_*2*_	*S*_*3*_	*S*_*4*_	*S*_*5*_
D_1_	1.62	3.32	5.45	9.72	11.49
D_2_	3.22	1.11	3.16	7.29	8.67
D_3_	4.97	1.49	1.59	5.95	7.71
D_4_	6.25	2.55	0.62	4.47	5.91
D_5_	7.84	3.93	1.11	3.24	4.55
D_6_	10.83	7.41	4.50	0.77	1.37
D_7_	8.87	5.48	2.73	3.52	4.71
D_8_	9.98	6.63	3.78	5.17	6.17

### Model formulation

In this section, a case study of maritime emergency resource allocation in Shandong illustrates the applicability of the proposed robust optimization model.

In this case study, we just focus on the oil dispersant allocation. As for only one kind of emergency resource, the robust optimization model of oil dispersant allocation in Shandong can be simplified as the following model.

$$\begin{array}{l} f_{1}=\min\left\{\sum_{i\in I}d_{i}^{0}\left(\sum_{j\in J}t_{ij}\omega_{ij}\right)+\Gamma_{0}\gamma +\sum_{i\in J_{0}}\lambda_{i}\right\}\\ f_{2}=\min\{\sum_{j\in J}x_{j}+\sum_{i\in I}d_{i}^{0}\left(\alpha \varpi_{i}-\sum_{j\in J}\omega_{ij}\right)+\Gamma_{0}\gamma^{'}+\sum_{i\in J_{0}}\lambda_{i}^{'}\}\\ s.t.\left\{ \begin{array}{l} x_{j}\leq V_{j},\forall j\\ \sum_{i\in I}\omega_{ij}d_{i}^{0}+\Gamma_{0}\varsigma_{j}+\sum_{i\in J_{0}}\tau_{ij}\leq x_{j},\forall j\\ \sum_{j\in J}\omega_{ij}+\varpi_{i}\geq 1,\forall i\\ \varsigma_{j}+\tau_{ij}\geq {\hat{d}}_{i}\omega_{ij}\forall i\in J_{0}\forall j\\ \gamma +\lambda_{i}\geq {\hat{d}}_{i}\left(\sum_{j\in J}t_{ij}\omega_{ij}\right)\forall i\in J_{0}\\ \gamma^{'}+\lambda_{i}^{'}\geq {\hat{d}}_{i}\left(\alpha \varpi_{i}-\sum_{j\in J}\omega_{ij}\right)\forall i\in J_{0}\\ x_{j},\omega_{ij},\varpi_{i},\varsigma_{j},\tau_{ij},\gamma ,\lambda_{i},\gamma^{'},\lambda_{i}^{'}\geq 0,\forall i,j \end{array}\right. \end{array}$$ (20)

The reliability analysis of robust solution is depicted in Supporting information *S2* Appendix, the probability that the robust solution violating the constraint is less than $\exp\left(-\Gamma_{0}^{2}/2\left|J_{0}\right|\right)$, in other words, the reliability of robust solution is more than $1-\exp\left(-\Gamma_{0}^{2}/2\left|J_{0}\right|\right)$.

### Model testing

Put the dynamic demand (Table 9) and the transportation time (Table 11) into the model (20), define the capacity of supplier center as *V* = 300, the penalty coefficient of demand *α* = 2. The NSGA-II algorithm is introduced to solve the robust optimization model of oil dispersant allocation in Shandong by matlab7.0. The population size is 50, the maximum evolutionary algebra is 30, the mutation rate is 0.001, the crossover probability is 0.9, in the case that the level of demand disturbance is of 5%, 10%, 20% respectively, when the robust control parameters change between 0 and 8, the program runs 24 times, with an average running time of 245s.

**Fig 4:**
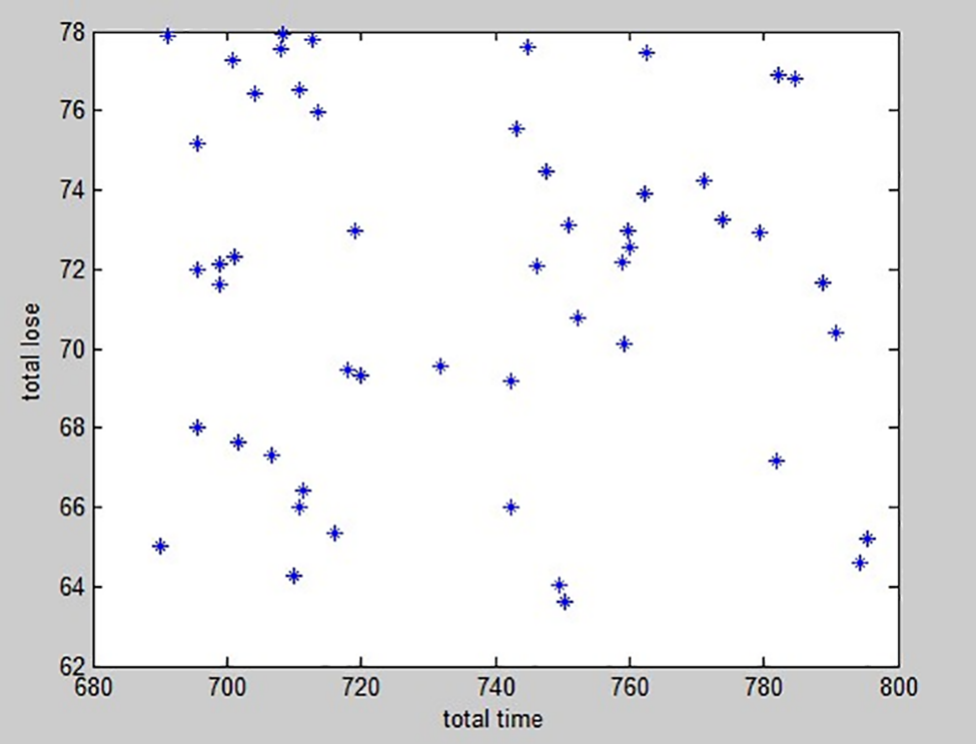
Distribution of optimal solution.

By the robust optimization approach, the allocation plan performs well under the various situation of dynamic demand. When the disturbance level of 10% and the robust control parameter of 4, the distribution of the feasible solution of the emergency resource allocation is shown in Fig 4.When the population evolves to the last generation, the distribution of the visible solution is seen rather uniform, The global search results are good.

There are two objectives in the emergency resource allocation optimal optimization model of the case. With the demand disturbance and robust control parameters changing, both of the total time and loss may not be optimal at the same time, indicating the optimal solution may not exist. In this case, the optimality of the total loss is given priority, the results of optimized oil dispersant schedule is shown in Tables 12–14, when the demand disturbance level is of 5%, 10%, 20% respectively. Due to the auxiliary decision variables in the model belong to the intermediate variables during the model solving, serving for the final decision variables, there is no need to list them.

**Table 12:** The optimized oil dispersant schedule under 5% demand disturbance.

Schedule Robust control parameters	Reserve stock(tons)					Objective	
	*x*_*1*_	*x*_*2*_	*x*_*3*_	*x*_*4*_	*x*_*5*_	*f*_*1*_(tons hours)	*f*_*2*_(tons)
Γ = 0	51	150	205	81	0	635	0
Γ = 1	51	156	207	83	1	633	13
Γ = 2	51	154	208	82	2	640	17
Γ = 3	52	155	209	83	2	682	18
Γ = 4	52	155	209	84	2	683	13
Γ = 5	52	156	211	84	3	712	25
Γ = 6	52	154	213	84	3	725	35
Γ = 7	53	156	212	85	3	741	28
Γ = 8	53	155	214	85	3	764	32

**Table 13:** The optimized oil dispersant schedule under 10% demand disturbance.

Schedule Robust control parameters	Reserve stock(tons)					Objective	
	*x*_*1*_	*x*_*2*_	*x*_*3*_	*x*_*4*_	*x*_*5*_	*f*_*1*_(tons hours)	*f*_*2*_(tons)
Γ = 0	51	150	205	81	0	635	0
Γ = 1	53	155	213	81	4	642	21
Γ = 2	53	161	217	82	3	663	32
Γ = 3	53	153	215	87	5	687	43
Γ = 4	54	158	217	86	7	690	65
Γ = 5	56	155	219	83	6	735	62
Γ = 6	52	153	215	82	4	760	54
Γ = 7	53	162	218	88	6	797	78
Γ = 8	54	152	224	85	6	804	75

**Table 14:** The optimized oil dispersant schedule under 20% demand disturbance.

Schedule Robust control parameters	Reserve stock(tons)					Objective	
	*x*_*1*_	*x*_*2*_	*x*_*3*_	*x*_*4*_	*x*_*5*_	*f*_*1*_(tons hours)	*f*_*2*_(tons)
Γ = 0	51	150	205	81	0	635	0
Γ = 1	59	162	236	94	3	614	32
Γ = 2	56	157	226	93	6	686	45
Γ = 3	59	173	240	89	8	735	76
Γ = 4	58	177	234	92	9	802	104
Γ = 5	56	177	225	90	4	745	133
Γ = 6	57	173	235	87	11	831	156
Γ = 7	55	177	240	92	13	867	132
Γ = 8	60	168	243	93	14	843	151

As is mentioned above, the robust control parameter Γ is the number of perturbation demand, indicating the risk preference of the decision maker. The greater of Γ, the more conservative of the decision maker in the face of the demand disturbance. On the contrary, that is more adventure. Γ is changing from 0 to 8. When Γ = 0, all the demand of the disaster areas is certain, as the nominal demand value, then the robust optimization model is considered as a general deterministic emergency resource allocation model. When Γ = 8, all the demand of the disaster areas is dynamic, it is the most conservative for emergency Resource allocation. Usually, the oil dispersant demand of some disaster area are inclined to fluctuate, and the others are keeping in the nominal demand, the robust optimized oil dispersant schedule can minimize the of total time and loss as much as possible, not always an optimal solution, a feasible solution basically. The robust optimization solution is analyzed as follows.

(1) Reliability analysis of robust optimization solution

As the S1 Appendix demonstrated, the reliability formula of oil dispersant solution for robust optimization allocation is $P(\Gamma_{0})>1-\exp\left(-\Gamma_{0}^{2}/2\left|J_{0}\right|\right)$. Therefore, with the robust control parameters fluctuating in the range of 1–8, the reliability of robust optimized solution can be shown in Table 15.

**Table 15:** The reliability of robust optimized oil dispersant solution.

Γ	0	1	2	3	4	5	6	7	8
Lower limit of reliability (%)	2.01	6.06	22.12	43.02	63.21	79.04	89.46	95.32	98.17

It can be seen that, under the dynamic demand condition, when the robust control parameter is more than 5, the reliability of solution is above 80%, and the robust optimization solution can guarantee the effective probability of the resource allocation model. The reliability of the certain allocation is the worst, which can not response to the change of the dynamic demand of the disaster area flexible.

(2) Comparison of robust optimal scheme and definite scheme

It’s easy to find that, from Tables 12–14, comparing with the definite resource allocation, under the demand disturbance level of 5%, 10% and 20%, the total quantity of the oil dispenser and total time and loss are increasing. The total time and loss are increases by 129 tons and 35 tons when the demand disturbance is 5%. So, the ability to response to demand disturbance is obtained at the expense of total time and loss increasing.

(3) Analysis of robust optimal allocation

**Fig 5:**
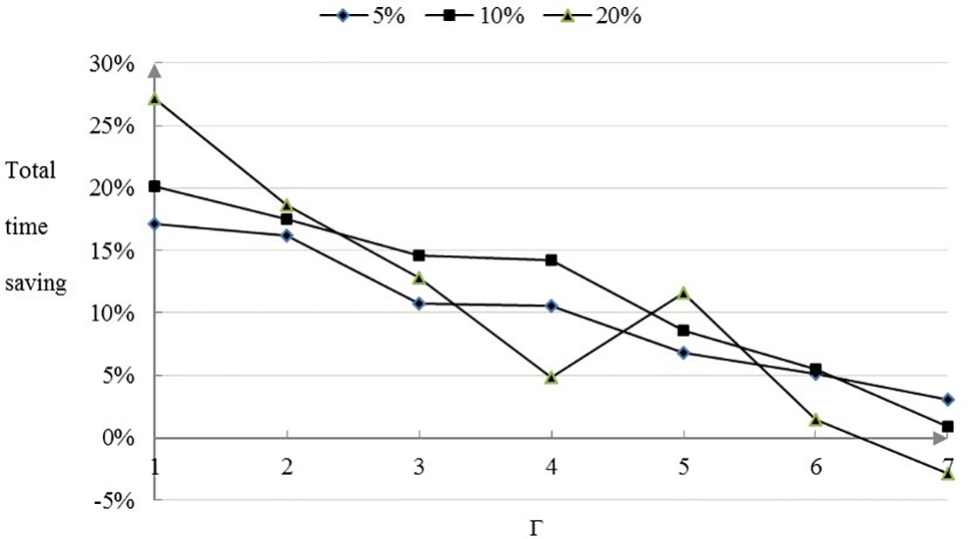
The proportion of total time saving.

**Fig 6:**
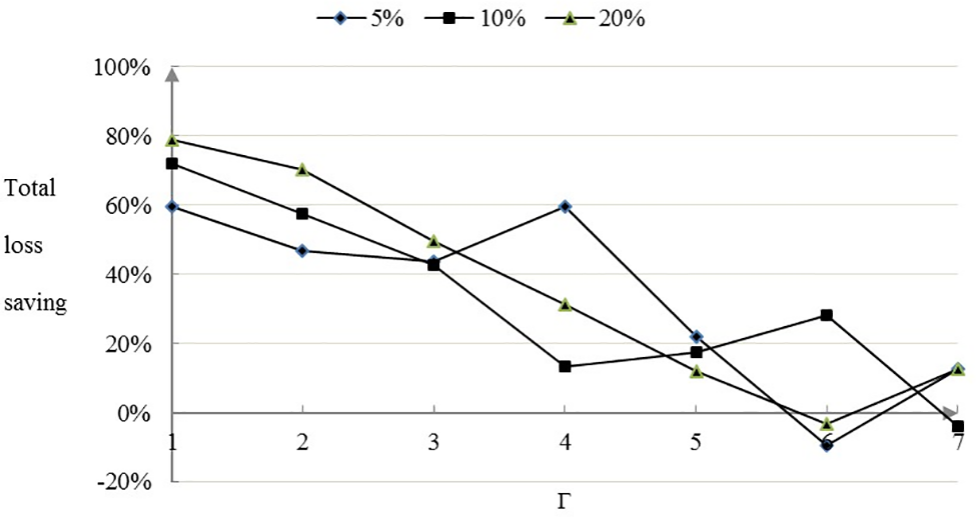
The proportion of total loss saving.

The more conservative the decision maker attitude is in the face of the demand disturbance, that is, the greater value of the robust control parameter Γ, the greater of the total time and loss of the robust optimized scheme. Compared with the most conservative oil dispersant allocation scheme, when the demand disturbance level is 5%, 10% and 20%, the robust control parameter Γ is 1–7,the proportion of total time and loss saving is shown in Figs 5 and 6 for the robust optimized oil dispersant scheme.

As is shown in Figs 5 and 6, with the increase of robust control parameters, the overall configuration savings ratio can be reduced by the optimal configuration scheme. In terms of total allocation time, there is a downward trend after rise. When the robust control parameter Γ is 1–5, the advantage of total time and loss saving is especially significant. When the robust control parameter Γ is 6,7, the total loss of the oil dispersant configuration is increased slightly. Compared with the most conservative oil spill dispersant configuration scheme, the robust optimal configuration scheme can save the total time proportion of the configuration is 27.86%, and the maximum cost of the configuration can save 78.80%. It can be seen that the effect is obvious that optimal optimization scheme can bring about the optimization in overall.

(4) The total quantity of oil dispenser

**Fig 7:**
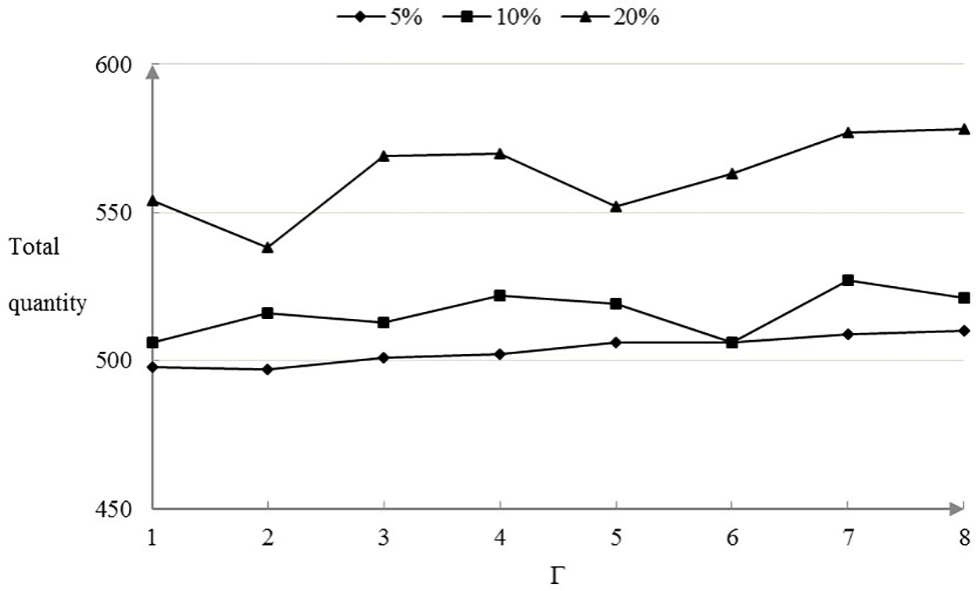
The total quantity of oil dispenser.

The total optimal reserves of the oil dispenser in the Shandong maritime area is the total stock of the oil dispenser in all supplier center. Under the condition that the demand disturbance is 5%, 10% and 20%, the optimal arrangement of the oil dispersant is optimized. The trend that the total quantity of oil dispenser changing with the robust control parameters is shown in Fig 7.

With the increase of the demand disturbance, in order to cope with the higher level of demand disturbance, the total quantity of oil spill dispersant is obviously increased. However, the variation law between the total quantity of the oil spill dispersant with the robust control parameters is not obvious. Due to the data characteristics of the dynamic demand distribution and the transportation time of the oil spill dispersant configuration robust optimization model, the stability of the oil spill dispersant configuration system is strong, and the total configuration loss and the minimum time can be realized when the total quantity of the allocation is basically stable. When the demand disturbance level is 5%, 10% and 20%, The total quantity of oil dispersive fluctuated around 503,516,562 tons generally.

Based on the above analysis of the robust optimization scheme of the oil dispersant in Shandong maritime area, we can get the following findings.

(1) The three important emergency resource supplier centers, that is Yantai, Weihai and Qingdao, are located in the middle of Shandong sea area. The location advantage is obvious, with the average distance is rather short to each accident area. So the reserve stocks of them are high. The robust optimized oil dispersant allocation scheme is reasonable, with the large emergency resource supplier center playing a leading role, the small emergency resource supplier center playing an auxiliary role. The result is in accordance with the actual allocation rules of emergency resources in China.

(2) Compared with the certain emergency resource allocation scheme, the robust optimal allocation scheme can cope with the demand disturbance, the reliability is strong and the optimization scheme is rigorous. Compared with the most conservative emergency resource allocation scheme, the robust optimal allocation scheme is more optimal in total loss and time saving, it is significant for the application in practical.

(3) With the increase of demand disturbance level, in order to response to the high level of demand disturbance, the additional resources to be allocated is also increased, However, the variation law between the total quantity of the oil spill dispersant with the robust control parameters is not obvious.

(4) The total quantity of the spill dispersant is not positively correlated with the allocation effectiveness. Robust control parameter Γ is the indicator measuring the conservation of decision maker. The more conservative of the decision maker, the stronger the reliability of the emergency resource allocation scheme, the weaker of the emergency resource allocation scheme.

Therefore, the decision makers should determine the reasonable robust control parameters according to their risk preference, seeking for balance between the robustness of the allocation scheme and the risk tolerance.

## Conclusions

This paper studies the dynamic demand, and define the dynamic demand with uncertain set. Then a maritime emergency resource allocation with uncertain data is presented. Afterwards, a robust approach is developed and used to make sure that the resource allocation schedule performs well when the data changes. At last a case study is given, we flexibly adjust the level of conservatism of the robust solutions in terms of probabilistic bounds of constraint violations, and draw some interesting conclusions.

(1) The maritime dynamic demand analysis is divided into three stages, dynamic demand forecasting, disaster area grouping and dynamic demand distribution. An efficient method is proposed in disaster grouping, combining the K-means clustering method with minimum circle coverage, overcoming the blindness of the traditional K-means clustering method and the initial clustering center randomly selected method.

(2) Considering the dynamic demand of maritime emergency resource, a robust optimization model is built up for emergency resource allocation. By the model solution, the optimal stock of emergency resource is determined for supplier centers according to the risk preference and the demand disturbance given by decision makers before disaster occurrence.

(3) The case study shows that, compared with the certain emergency resource allocation scheme, the robust optimal allocation scheme could cope with the demand disturbance. The more conservative of decision maker, the stronger the reliability of the emergency resource allocation scheme. However, the total quantity of the emergency resource is not positively correlated with the allocation effectiveness.

The proposed methodology is feasible in maritime emergency resource allocation. The findings could help emergency manager schedule the emergency resource allocation flexibly in terms of dynamic demand, regarding to their risk preferences.

Nevertheless, there is still a great potential for improving the study of maritime emergency resource allocation. Firstly, the dynamic demand forecasting approach is limited to the some type of maritime emergency resource, not covering all kind of emergency resource. Secondly, the transportation time for emergency resource may change due to the maritime environment. Furthermore, the algorithm for the model solving is designed by matlab, the more advanced technology can be incorporated. The further study is particular important to carry out the ultimate goals of maritime emergency logistics management.

## Supporting information

S1 Appendix Reliability of robust optimization solution. S1 Appendix presents the derivation process of the robust optimization solution reliability.
(DOCX)

S2 Appendix The details of maritime accidents in Shandong maritime region. S2 Appendix list the details of the maritime accidents in Shandong maritime region, including the time, number of casualties, number of damaged ships for period 2011–2015.
(DOCX)
