# Supplementary material for: Optimized maritime emergency resource allocation under dynamic demand
Source: PLoS One. 2017 Dec 14;12(12):e0189411. doi: 10.1371/journal.pone.0189411 (PMC5730157; doi:10.1371/journal.pone.0189411)
Supplement: S1 Appendix — presents the derivation process of the robust optimization solution reliability. (DOCX) [file pone.0189411.s001.docx]

**Supporting information**

**S_1_ Appendix**

Table 1 Details of Maritime accidents in Shandong Maritime Region

| Order number of accident | time | Coordinates (E,N) | The number of casualties | The number of damaged ships |
| --- | --- | --- | --- | --- |
| 1 | 20151118 | (119.16,38.05) | 7 | 1 |
| 2 | 20151117 | (119.26,37.87) | 9 | 1 |
| 3 | 20151104 | (120.67,35.15) | 5 | 1 |
| 4 | 20151021 | (118.84,38.21) | 6 | 1 |
| 5 | 20150928 | (119.59,35.31) | 11 | 1 |
| 6 | 20150807 | (122.03,37.92) | 2 | 0 |
| 7 | 20150818 | (122.12,37.49) | 4 | 1 |
| 8 | 20150811 | (122.54,36.78) | 1 | 0 |
| 9 | 20150803 | (119.96,38.22) | 1 | 0 |
| 10 | 20150802 | (121.94, 37.46) | 1 | 0 |
| 11 | 20150713 | (122.37, 36.87) | 28 | 1 |
| 12 | 20150606 | (120.18, 35.87) | 5 | 1 |
| 13 | 20150413 | (123.48, 35.05) | 1 | 0 |
| 14 | 20150405 | (122.72, 36.65) | 14 | 2 |
| 15 | 20150322 | (118.91, 38.15) | 9 | 1 |
| 16 | 20150311 | (122.14, 38.00) | 10 | 2 |
| 17 | 20150222 | (121.76, 37.46) | 8 | 1 |
| 18 | 20150211 | (122.72, 36.42) | 1 | 0 |
| 19 | 20150131 | (122.44, 35.26) | 14 | 1 |
| 20 | 20150112 | (124.28, 35.45) | 1 | 0 |
| 21 | 20150106 | (120.88, 37.9) | 6 | 1 |
| 22 | 20141218 | (122.45, 36.86) | 8 | 1 |
| 23 | 20141207 | (123.80, 35.42) | 22 | 1 |
| 24 | 20141207 | (122.44, 35.42) | 1 | 0 |
| 25 | 20141206 | (123.55, 35.98) | 1 | 0 |
| 26 | 20141203 | (121.94, 37.79) | 8 | 1 |
| 27 | 20141201 | (120.76, 37.86) | 10 | 1 |
| 28 | 20141127 | (122.81, 37.39) | 4 | 1 |
| 29 | 20141127 | (122.85, 36.59) | 10 | 2 |
| 30 | 20141030 | (120.18, 35.75) | 6 | 1 |
| 31 | 20140904 | (122.85, 36.59) | 1 | 0 |
| 32 | 20140902 | (121.07, 37.99) | 1 | 0 |
| 33 | 20140813 | (122.75, 37.27) | 1 | 0 |
| 34 | 20140808 | (120.72,35.79 ) | 1 | 0 |
| 35 | 20140804 | (122.85, 36.86) | 43 | 2 |
| 36 | 20140722 | (121.62 ,37.84) | 2 | 2 |
| 37 | 20140612 | (121.84, 36.04) | 3 | 1 |
| 38 | 20140525 | (122.18, 36.67) | 5 | 1 |
| 39 | 20140521 | (122.76,36.04) | 21 | 2 |
| 40 | 20130201 | (120.31,36.03) | 23 | 2 |
| 41 | 20130603 | (121.48,38.13) | 8 | 1 |
| 42 | 20130513 | (120.23,37.63) | 2 | 1 |
| 43 | 20130405 | (119.25,37.28) | 5 | 1 |
| 44 | 20130416 | (122.78,37.32) | 9 | 1 |
| 45 | 20130322 | (118.98,37.57) | 3 | 1 |
| 46 | 20130308 | (122.15,36.60) | 7 | 2 |
| 47 | 20130224 | (121.22,36.00) | 16 | 1 |
| 48 | 20130121 | (121.41, 37.57) | 20 | 1 |
| 49 | 20130117 | (123.23,35.60) | 14 | 1 |
| 50 | 20121120 | (122.59,38.20) | 10 | 1 |
| 51 | 20121019 | (123.80,35.50) | 1 | 0 |
| 52 | 20120802 | (120.35,36.18) | 8 | 2 |
| 53 | 20120707 | (122.61,37.14) | 2 | 2 |
| 54 | 20120601 | (122.44,36.36) | 1 | 0 |
| 55 | 20120515 | (122.49,36.38) | 9 | 2 |
| 56 | 20120512 | (122.50,35.93) | 1 | 0 |
| 57 | 20120510 | (123.32,37.27) | 1 | 0 |
| 58 | 20120503 | (120.32,35.35) | 1 | 0 |
| 59 | 20120426 | (120.90,38.13) | 14 | 1 |
| 60 | 20120420 | (124.47, 36.50) | 1 | 0 |
| 61 | 20120331 | (120.24,37.62) | 7 | 1 |
| 62 | 20120330 | (124.37,36.05) | 1 | 0 |
| 63 | 20120323 | (122.75,37.87) | 18 | 1 |
| 64 | 20120304 | (119.93,38.07) | 1 | 0 |
| 65 | 20120302 | (123.53,35.73) | 1 | 0 |
| 66 | 20120219 | (122.36, 37.63) | 1 | 0 |
| 67 | 20120218 | (120.94,35.88) | 1 | 0 |
| 68 | 20120125 | (122.18,37.70) | 1 | 0 |
| 69 | 20120108 | (122.58, 37.75) | 1 | 0 |
| 70 | 20111229 | (120.86, 37.93) | 1 | 0 |
| 71 | 20111226 | (119.67, 38.32) | 1 | 0 |
| 72 | 20111217 | (122.52, 37.78) | 0 | 2 |
| 73 | 20111229 | (122.82, 36.86) | 10 | 2 |
| 74 | 20111122 | (120.25, 37.63) | 4 | 1 |
| 75 | 20111128 | (118.51,38.20) | 7 | 1 |
| 76 | 20111102 | (121.28, 38.35) | 9 | 2 |
| 77 | 20111101 | (122.75, 36.42) | 7 | 1 |
| 78 | 20111028 | (120.43, 35.98) | 19 | 2 |
| 79 | 20111028 | (123.83, 35.15) | 1 | 0 |
| 80 | 20111024 | (122.95, 36.75) | 12 | 1 |
| 81 | 20111021 | (123.98, 38.08) | 1 | 0 |
| 82 | 20111020 | (124.23, 34.92) | 1 | 0 |
| 83 | 20111017 | (122.25, 35.60) | 1 | 0 |
| 84 | 20111017 | (124.27,35.12) | 1 | 0 |
| 85 | 20111009 | (122.68, 37.57) | 1 | 0 |
| 86 | 20111009 | (121.22, 38.40) | 9 | 2 |
| 87 | 20110917 | (120.92,38.30) | 11 | 1 |
