# Supplementary material for: Optimized maritime emergency resource allocation under dynamic demand
Source: PLoS One. 2017 Dec 14;12(12):e0189411. doi: 10.1371/journal.pone.0189411 (PMC5730157; doi:10.1371/journal.pone.0189411)
Supplement: S2 Appendix — list the details of the maritime accidents in Shandong maritime region, including the time, number of casualties, number of damaged ships for period 2011–2015. (DOCX) [file pone.0189411.s002.docx]

**Supporting information**

**S_2_ Appendix**

It is clear by the construction of the robust formulation that if up to $\Gamma_{0}$of uncertain demand change with their bounds,the solution of the robust optimization model will remain fesasile. In this section,we will study the solution validation when the dynamic demand takes value in the interval$(d_{mi}^{0}-\hat{d}_{mi},d_{mi}^{0}+\hat{d}_{mi})$,which is not controlled by$\Gamma_{m}$or$\Gamma_{0}$.

Let $\omega_{mij}^{*}$，$U_{m}^{*}$，$r_{m}^{*}$，$x_{mj}^{*}$be the solution of the model(17),we can get:

$$\Pr\left(\sum_{i\in I} \omega_{mij}^{*}d_{mi}>x_{mj}^{*}\right)\leq\Pr(\sum_{i\in I} \chi_{mi}\eta_{mi}\geq\Gamma_{m})\leq\exp\left(-\frac{\Gamma_{m}^{2}}{2\left|J{}_{m}\right|}\right)$$

Where $\eta_{mi}=\frac{d_{mi}-d_{mi}^{0}}{\hat{d}_{mi}}$；$\chi_{mi}=\left\{\begin{matrix} 1\begin{matrix} & & \begin{matrix} & & i\in U_{m}^{*} \end{matrix} \end{matrix} \\ \sum_{j\in J} \frac{\hat{d}_{mi}\omega_{mij}^{*}}{\hat{d}_{mr_{m}^{*}}\omega_{mr_{m}^{*}j}^{*}}\begin{matrix} & i\in J_{m}\backslash U_{m}^{*} \end{matrix} \end{matrix}\right.$，$r_{m}^{*}=\mathrm{argmin}\hat{d}_{mr_{m}}\omega_{mr_{m}j}$

PROOF

$$\begin{matrix} \Pr\left(\sum_{i\in I} \omega_{mij}^{*}d_{mi}>x_{mj}^{*}\right) =\Pr\left(\sum_{i\in I} d_{mi}^{0}\omega_{mij}^{*}+\sum_{i\in J_{m}} \hat{d}_{mi}\omega_{mij}^{*}\eta_{mi}>x_{mj}^{*}\right) \\ \leq\Pr\left(\sum_{i\in J_{m}} \hat{d}_{mi}\omega_{mij}^{*}\eta_{mi}>\sum_{i\in U_{m}^{*}} \hat{d}_{mi}\omega_{mij}^{*}+(\Gamma_{m}-\left\lfloor\Gamma_{m}\right\rfloor)\hat{d}_{mr_{m}^{*}}\omega_{mr_{m}j}^{*}\right) \\ =\Pr\left(\sum_{i\in J_{m}\backslash U_{m}^{*}} \hat{d}_{mi}\omega_{mij}^{*}\eta_{mi}>\sum_{i\in U_{m}^{*}} \hat{d}_{mi}\omega_{mij}^{*}(1-\eta_{mi})+(\Gamma_{m}-\left\lfloor\Gamma_{m}\right\rfloor)\hat{d}_{mr_{m}^{*}}\omega_{mr_{m}^{*}j}^{*}\right) \\ \leq\Pr\left(\sum_{i\in J_{m}\backslash U_{m}^{*}} \hat{d}_{mi}\omega_{mij}^{*}\eta_{mi}>\hat{d}_{mr_{m}^{*}}\omega_{mr_{m}^{*}j}^{*}(\sum_{i\in U_{m}^{*}} (1-\eta_{mi})+\Gamma_{m}-\left\lfloor\Gamma_{m}\right\rfloor)\right) \\ =\Pr\left(\sum_{i\in U_{m}^{*}} \eta_{mi}+\sum_{i\in J_{m}\backslash U_{m}^{*}} \sum_{j\in J} \frac{\hat{d}_{mi}\omega_{mij}^{*}}{\hat{d}_{mr_{m}^{*}}\omega_{mr_{m}^{*}j}^{*}}\eta_{mi}>\Gamma_{m}\right) \\ =\Pr\left(\sum_{i\in J_{m}} \chi_{mi}\eta_{mi}>\Gamma_{m}\right) \\ \leq\Pr\left(\sum_{i\in J_{m}} \chi_{mi}\eta_{mi}\geq\Gamma_{m}\right) \end{matrix}$$

$$\begin{matrix} \Pr\left(\sum_{i\in J_{m}} \chi_{mi}\eta_{mi}\geq\Gamma_{m}\right) \leq\frac{E[\exp(\theta\sum_{i\in J_{m}} \chi_{mi}\eta_{mi})]}{\exp(\theta\cdot\Gamma_{m})} \\ =\frac{\prod_{i\in J_{m}} E[\exp(\theta\chi_{mi}\eta_{mi})]}{\exp(\theta\cdot\Gamma_{m})} \\ =\frac{\prod_{i\in J_{m}} 2\int_{0}^{1} \sum_{k=0}^{\infty} (\theta\chi_{mi}\eta)^{2k}/(2k)!dF_{\eta_{mi}}(\eta)}{\exp(\theta\cdot\Gamma_{m})} \\ \leq\frac{\prod_{i\in J_{m}} \sum_{k=0}^{\infty} (\theta\chi_{mi})^{2k}/(2k)!}{\exp(\theta\cdot\Gamma_{m})} \\ \leq\frac{\prod_{i\in J_{m}} \exp(\theta\chi_{mi})^{2}/2}{\exp(\theta\cdot\Gamma_{m})} \\ \leq\exp(\left|J_{i}\right|\frac{\theta^{2}}{2}-\theta\cdot\Gamma_{m}) \end{matrix}$$

Selecting $\theta=\Gamma_{m}/\left|J_{m}\right|$，we can obtain：$\Pr(\sum_{i\in I} \chi_{mi}\eta_{mi}\geq\Gamma_{m})\leq\exp\left(-\Gamma_{m}^{2}/2\left|J{}_{m}\right|\right)$.So the validation of optimal solution for marine emergency resource allocation is more then$1-\exp\left(-\Gamma_{m}^{2}/2\left|J{}_{m}\right|\right)$.
